# Identification of antitumoral agents against human pancreatic cancer cells from Asteraceae and Lamiaceae plant extracts

**DOI:** 10.1186/s12906-018-2322-6

**Published:** 2018-09-17

**Authors:** Lamia Mouhid, Marta Gómez de Cedrón, Teodoro Vargas, Elena García-Carrascosa, Nieves Herranz, Mónica García-Risco, Guillermo Reglero, Tiziana Fornari, Ana Ramírez de Molina

**Affiliations:** 1 Molecular Oncology and Nutritional Genomics of Cancer, Madrid Institute for Advanced Studies on Food (IMDEA-Food), Ctra de Cantoblanco, 8, 28049 Madrid, Spain; 2 Production and Characterization of Novel Foods Department, Institute of Food Science Research (CIAL) CEI UAM + CSIC, Madrid, Spain

**Keywords:** Pancreatic cancer, Asteraceae, Lamiaceae, Chemotherapeutic agents, 5-Fluororacil

## Abstract

**Background:**

Pancreatic cancer is one of the most aggressive and mortal cancers. Although several drugs have been proposed for its treatment, it remains resistant and new alternatives are needed. In this context, plants and their derivatives constitute a relevant source of bioactive components which might efficiently inhibit tumor cell progression.

**Methods:**

In this study, we have analyzed the potential anti-carcinogenic effect of different Asteraceae (*Achillea millefolium* and *Calendula officinalis*) and Lamiaceae (*Melissa officinalis* and *Origanum majorana*) plant extracts obtained by different green technologies (Supercritical CO_2_ Extraction –SFE- and Ultrasonic Assisted Extraction –UAE-) to identify efficient plant extracts against human pancreatic cancer cells that could constitute the basis of novel treatment approaches.

**Results:**

Asteraceae extracts showed better results as antitumoral agents than Lamiaceae by inducing cytotoxicity and inhibiting cell transformation, and SFE extracts were most efficient than UAE extracts. In addition, SFE derived plant extracts from *Achillea millefolium* and *Calendula officinalis* displayed synergism with the chemotherapeutic 5-Fluororacil.

**Conclusion:**

These results show how Yarrow and Marigold SFE-derived extracts can inhibit pancreatic cancer cell growth, and could be proposed for a comprehensive study to determine the molecular mechanisms involved in their bioactivity with the final aim to propose them as potential adjuvants in pancreatic cancer therapy.

**Electronic supplementary material:**

The online version of this article (10.1186/s12906-018-2322-6) contains supplementary material, which is available to authorized users.

## Background

As reported by the European Society for Medical Oncology (ESMO), pancreatic cancer is the seventh most common cancer in Europe, accounting for 2.8% of cancer in men and 3.2% in women. It is the fifth leading cause cancer-related death with 70,000 estimated deaths each year [1]. Depending on the tumor stage and if surgery is possible, current treatments are based on the use of the antimetabolite 5-fluorouracil (5-FU), but also gemcitabine, nab-paclitaxel, or in combination with acid folic, such as Folfirinox (folic acid+ 5-FU or irinotecan or oxaliplatin) [2]. A reduced percentage of patients respond to these antitumor agents due to the advanced stage of the tumor when it is diagnosticated and due to the appearance of resistances. In this scenario, plants are an important source to obtain new compounds that could be used as chemotherapeutic drugs.

In the last decades, plant-derived compounds have been clinically used as anti-cancer agents [3, 4], as they demonstrate the ability to modulate several molecular pathways involved in tumor development and progression. In this sense, the expected intervention for a plant-derived extract as antitumoral agent should exert a cytotoxic effect in tumor cells, without affecting cell viability of normal cells.

In a recent work, we produced and chemically characterized several extracts obtained from Lamiaceae (*Melissa officinalis* or Balm and *Origanum majorana* or Marjoram) and Asteraceae (*Achillea millefolium* or Yarrow, and *Calendula officinalis* or Marigold) plant families. For this purpose, we produced two different extracts from each plant by applying two sequential extraction steps: first Supercritical Fluid Extraction (SFE) and then, the remained vegetal raw material was re-extracted by Ultrasonic Assisted Extraction (UAE) [5].

**Fig. 1:**
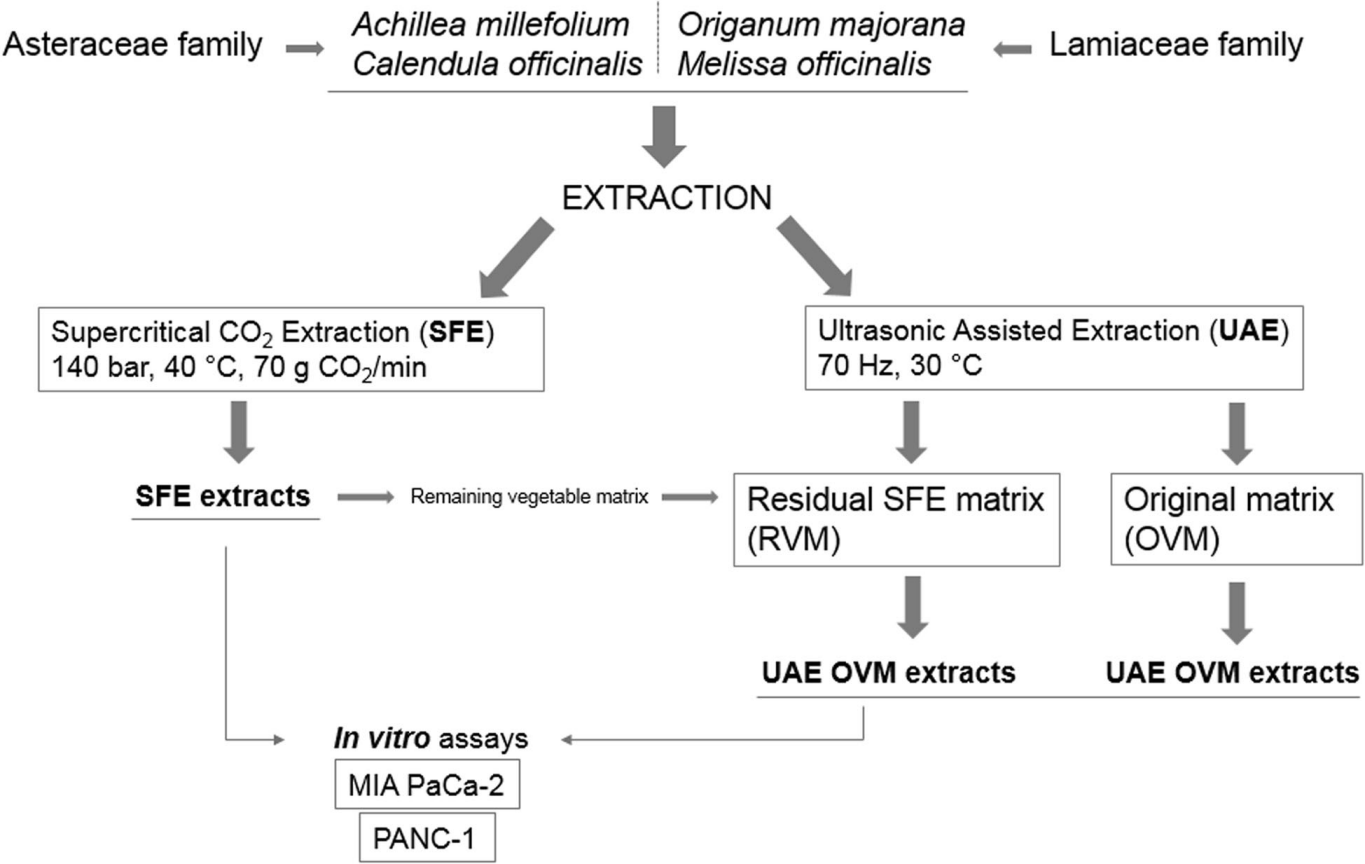
Screening overview and general approach addressed in the present work

Herein, in the present work, we investigated the antitumoral activity of those extracts obtained from Balm, Marjoram, Yarrow and Marigold SFE and UAE extracts in pancreatic cancer cell lines models (MIA PaCa-2 and PANC-1), and we have compared their biological activity and efficiency between the two extraction approaches (Fig. 1). Furthermore, additional UAE extracts were produced using the original vegetal matrix (OVM) for comparison with those produced previously [4] using the SFE residual vegetal matrix (RVM).

Although previous works have been described the antitumoral properties exerted by Marjoram [6, 7], Balm [8, 9], Yarrow [10, 11], and Marigold [12, 13], to our knowledge, the novelty of the present work lies in the analysis of extracts produced by environmentally friendly extraction techniques together with advanced biological methods in order to obtain, test and characterize natural extracts that could be potentially used for cancer therapies. After identifying the most efficient extracts inducing pancreatic cells toxicity, we further studied the mechanisms through which they induce cell death (apoptosis or necrosis) and their potentiality to inhibit cell malignancy and invasion. We also have evaluated the putative synergism with 5-FU to propose an efficient antitumoral product as a potential adjuvant in pancreatic cancer treatment. Our findings indicate that the SFE extracts obtained from Yarrow and Marigold meet the requirements to be proposed as a promising antitumoral approach.

## Methods

### Reagents

DMEM (Dulbecco’s Modified Eagle Medium), PBS (Phosphate-buffered saline), glutamine and trypsin were purchased from Lonza Spain; and FBS from Thermo Fisher Scientific. DMSO (Dimethyl Sulfoxide) and Ethanol were purchased from Scharlab S.L. MTT (3-(4,5-dimethylthiazol-2-yl)-2,5-diphenyltetrazolium bromide) and Staurosporine were purchased from Sigma Aldrich. Plants leaves for producing the extracts were purchased from Herboristería Murciana (Murcia, Spain), and commercial extracts were purchased from Soria Natural, S.A.

### Extraction procedures

Extracts were obtained by mean of two different green technologies as described previously [[5]]. Briefly, SFE was carried out using supercritical CO_2_ (140 bar, 40 °C, 70 g CO_2_/min), and UAE derived extracts using ethanol or ethanol: water − 50:50- (70 Hz, 30 °C). In addition, and regarding UAE, two different extracts were compared in the present work: the extracts produced from the Original Vegetable Matrix (OVM) and the those produced from the Residual Vegetable Matrix (RVM) after the SFE extraction. Fig. 1 shows a schematic overview of all the extracts evaluated in this study.

Chemical composition of the extracts is described in a previous work [4]. The extracts obtained by ultrasonic probe are rich in flavonoids and phenols, whereas those obtained by supercritical CO_2_ are rich in sesquiterpenes and monoterpenes, which were detailed by a gas-chromatography analysis [4].

### Cell culture

Human pancreatic cancer cells MIA PaCa-2 and PANC-1, obtained from American Type Culture Collection (ATCC), were cultured in DMEM supplemented with 10% FBS. Cells were kept under standard conditions of temperature (37 °C), humidity (95%) and carbon dioxide (5%).

For tridimensional culture, MIA PaCa-2 cells were grown in Matrigel Growth Factor Reduced (Corning® Life Sciences) 80%, and media was renewed every two days, until the 3D-spheres were formed. Treatments with extracts were then applied for 72 h.

### Cell viability assay

The cytotoxic and antiproliferative activities of the different extracts in human pancreatic tumor cell lines were determined by MTT assay. Briefly, cells in the exponential growth phase were plated in 96-multiwell plates. After 24 h, media was replaced with 200 μL media containing serial concentrations of each extract (dissolved in DMSO) for 48 h. The number of viable cells was determined at time zero (control growth wells) and after treatments. To determine the number of viable cells, tetrazolium MTT salt solution (Sigma) (5 mg/mL in phosphate- buffered saline) was added for 3 h. Then, the formazan produced in each well was solubilized by adding 200 μL DMSO and measured using a spectrophotometer reader (λ = 560 nm) (Biochrom Asys UVM 340 Microplate Reader; ISOGEN). Parameters for 50% of cell viability inhibition (IC50), 50% of cell growth inhibition (GI50), total cell growth inhibition (TGI), and 50% of cell death (LC50) were calculated accordingly to NIH definitions using a logistic regression [14].

The synergism between 5-FU and Yarrow and Marigold SFE extracts was analyzed by the combination index (CI) obtained using the Calcusyn software (Biosoft), based on the Chou-Talalay method [15].

### Flow cytometry

After 24 h’ culture in DMEM, pancreatic cancer MIA PaCa-2 cells were treated with increasing concentrations of Yarrow and Marigold SFE extracts for 24 h. Marjoram SFE extract was used as a negative control and staurosporine 1.5 μM as a positive control for apoptosis. The Annexin V and Propidium iodide (PI) staining was carried out by using Annexin V-FITC Apoptosis Detection kit (Immunostep, Spain) accordingly to manufacturer’s instructions. Stained cells were conducted on a Cytomics FC500 (Beckam Coulter) cytometer. Early apoptosis was defined as Ann+/PI- cells, whereas Ann+/PI+ cells was defined as late apoptosis and Ann-/PI+ cells were considered as necrotic cells.

### Caspase activation assay

MIA Paca-2 cells were plated in 96-multiwell and treated for 48 h with increasing concentrations of the extracts. The activation of caspase 3 and caspase 7 was determined using the Caspase-Glo 3/7 assay kit (Promega), following manufacturer’s instructions.

### Statistical analysis

Results were analyzed by ANOVA non-parametric with Bonferroni post hoc tests. Data were represented as mean ± S.E.M of at least three independent experiments. Statistical differences were defined as *p* < 0,05. Statistical analysis was performed with Graph Pad Prim 6 statistical software.

## Results

### Effect of Asteraceae and Lamiaceae families plant derived extracts on MiaPaca-2 cell viability

**Fig. 2:**
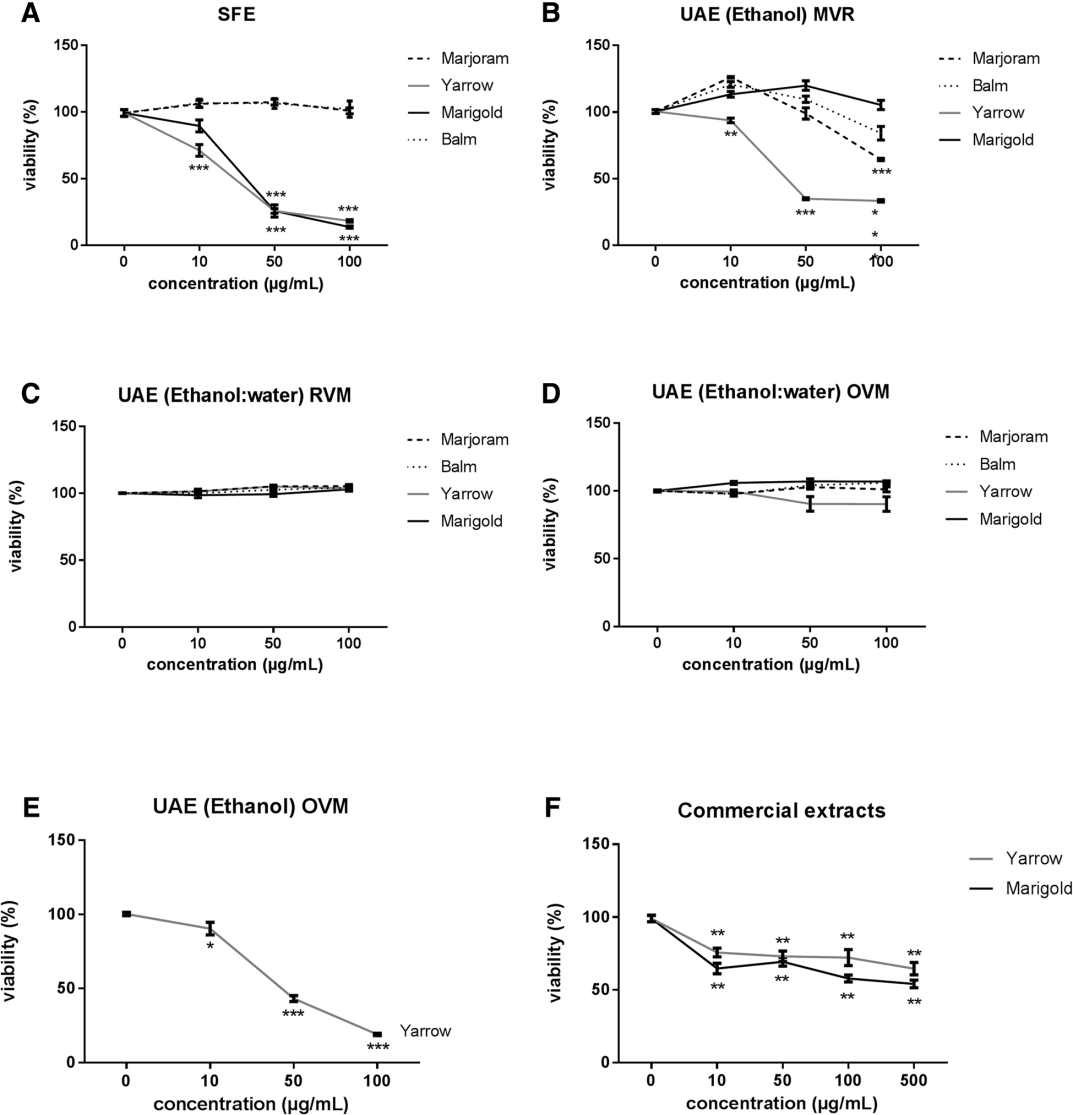
Dose-response curves of cell viability assays after 48 h treatment of MIA PaCa-2 human pancreatic cancer cells with increasing concentrations of SFE extracts (**a**), UAE (Ethanol) MVR extracts (**b**), UAE (Ethanol:Water) MVR extracts (**c**), UAE (Ethanol:Water) MVO extracts (**d**), UAE (Ethanol) MVO extracts (**e**) and commercial extracts (**f**). Data represent means ± S.E.M of at least three independent experiments each performed in quadruplicate. Asterisks indicate statistical differences in treated cells with respect to the control (non-treated cells, DMSO 0.1%). *p < 0.05; **p < 0.01; ***p < 0.001

We first determined the effect of all extracts on MIA PaCa-2 cell viability by MTT assay. In this assay, viable cells with active metabolism can convert MTT into a colored product by reducing the MTT into formazan [16]. MIA PaCa-2 cells were treated for 48 h with all extracts in a range of concentrations between 0 and 100 μg/ml. As observed in Fig. 2a, Yarrow and Marigold SFE extracts decreased cell viability in a dose-dependent manner (IC50 = 31,5 ± 8,6 μg/mL and 39,8 ± 4,6 μg/mL respectively [5]), while Balm and Marjoram SFE extracts did not affected cell viability at any of the doses tested.

Regarding UAE (Ethanol), Yarrow extracts, both obtained from RVM and from OVM, inhibited cell proliferation in a higher manner than the rest of the UAE plant extracts (Marigold, Marjoram and Balm) (Fig. 2b, e). We also checked UAE extracts obtained with a mix of ethanol: water (50:50) as solvent from both vegetal matrices (OVM, RVM), but none of them affected cell viability in any of the tested doses (Fig. 2c, d). Table 1 summarizes concentrations corresponding to 50% cell viability inhibition (IC50), 50% cell growth inhibition (GI50), total cell growth inhibition (TGI), and 50% of cell death (LC50) for all the extracts tested.

**Table 1:** Concentration parameters of the selected plant extracts on MIA PaCa-2 cell line sensitivity depending on the extraction method

		Extraction Method					
		SFE	UAE				Commercial
	Concentration Parameter		RVM		OVM		
Plant extract			Ethanol	Ethanol:Water	Ethanol	Ethanol:Water	
Marjoram	IC50	> 100^a^	> 100^a^	> 100^a^	–	> 100	–
	GI50	> 100	75,89 ± 2,05	> 100	–	> 100	–
	TGI	> 100	> 100	> 100	–	> 100	–
	LC50	> 100	> 100	> 100	–	> 100	–
Balm	IC50	> 100^a^	> 100^a^	> 100^a^	–	> 100	–
	GI50	> 100	> 100	> 100	–	> 100	–
	TGI	> 100	> 100	> 100	–	> 100	–
	LC50	> 100	> 100	> 100	–	> 100	–
Yarrow	IC50	31,4 ± 8,5^a^	48,5 ± 2,5^a^	> 100	28,8 ± 15,8	> 100	> 500
	GI50	37,4 ± 7,3	> 100	> 100	49,4 ± 25,7	> 100	> 500
	TGI	56 ± 1,4	65,1 ± 6,1	> 100	86,6 ± 3,4	> 100	> 500
	LC50	70,6 ± 7,1	> 100	> 100	> 100	> 100	> 500
Marigold	IC50	39,8 ± 4,6^a^	> 100^a^	> 100^a^	–	> 100	> 500
	GI50	–	> 100	> 100	–	> 100	> 500
	TGI	54, ± 3,1	> 100	> 100	–	> 100	> 500
	LC50	78,5 ± 1,4	> 100	> 100	–	> 100	> 500

Data (> 100): not significant activity found at 100 μg/mL concentration; (−): not determined; *SFE* Supercritical Fluid Extraction, *UAE* Ultrasound Assisted Extraction, *RVM* Residual Vegetable Matrix, *OVM* Original Vegetable Matrix. ^a^Data acquired from a previous work [4]

Data are presented as IC50 (μg/mL) (concentration needed to induce 50% cell viability inhibition [4, 5] <sup> 5</sup>, GI50 (μg/mL) (concentration needed for 50% growth inhibition), TGI (μg/mL) (concentration needed for total growth inhibition) and LC50 (μg/mL) (concentration needed for 50% cell death) after 48 h treatment as mean ± SEM of at least three independent experiments each performed in quadruplicate

With these preliminary results, SFE extracts from Asteraceae family (Marigold and Yarrow) seems to compromise cell viability by inducing lethal toxicity (Yarrow: LC50 = 70,6 ± 8,6 μg/mL and Marigold: LC50 = 78,5 ± 1,4 μg/mL) suggesting that these two extracts might act as promising antitumor agents against pancreatic cancer.

### Effect of yarrow and Marigold extracts on PANC-1 metastatic pancreatic cancer cell line

**Fig. 3:**
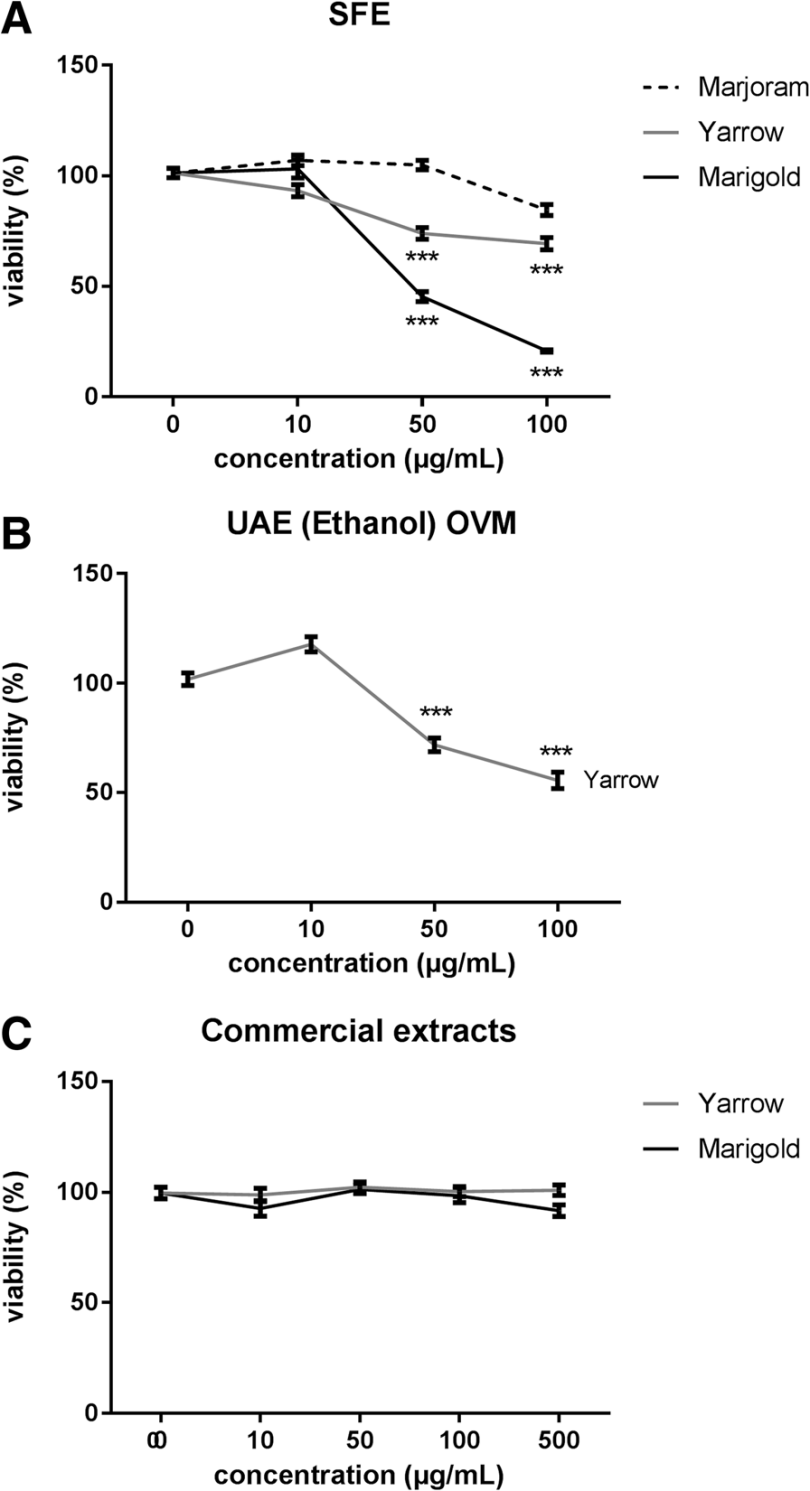
Dose-response curves of cell viability assays after 48 h treatment of PANC-1 pancreatic cancer cells with increasing concentrations of SFE extracts (**a**), UAE (Ethanol) MVO extracts (**b**) and commercial extracts (**c**) for PANC-1. Data represent means ± S.E.M of at least three independent experiments each performed in quadruplicate. Asterisks indicate statistical differences in treated cells with respect to the control (non-treated cells, DMSO 0.1%). ***p < 0.001

We next studied the effect of the most effective extracts from Asteraceae plants (Yarrow SFE and Marigold SFE) on PANC-1 pancreatic cancer cell line, described to be more resistant to treatments [14, 15]. As shown in Fig. 3a and Table 2, Marigold displayed the strongest cell growth inhibition (IC50 = 43,2 ± 7,9 μg/mL) and lethal effect (LC50 = 74,2 ± 6,6), while Yarrow did not exert any effect at the doses tested (IC50 >  100 μg/mL).

**Table 2:** Concentration parameters of the selected plant extracts on PANC-1 cell line sensitivity depending on the extraction method

		Extraction Method		
		SFE	UAE	Commercial
	Concentration Parameter		OVM	
Plant extract			Ethanol	
Marjoram	IC50	> 100	–	–
	GI50	> 100	–	–
	TGI	> 100	–	–
	LC50	> 100	–	–
Yarrow	IC50	> 100	> 100	> 500
	GI50	24,5 ± 0,26	> 100	> 500
	TGI	96,4 ± 6,2	69,2 ± 1,1	> 500
	LC50	> 100	> 100	> 500
Marigold	IC50	43,2 ± 7,9	–	> 500
	GI50	> 100	–	> 500
	TGI	45,9 ± 1,8	–	> 500
	LC50	74,2 ± 6,6	–	> 500

Data (> 100): not significant activity found at 100 μg/mL concentration; (−): not determined; *SFE* Supercritical Fluid Extraction, *UAE* Ultrasound Assisted Extraction, *OVM* Original Vegetable Matrix

Data are presented as IC50 (μg/mL) (concentration needed for 50% inhibition of cell proliferation), GI50 (μg/mL) (concentration needed for 50% growth inhibition), TGI (μg/mL) (concentration needed for total growth inhibition) and LC50 (μg/mL) (concentration needed for 50% cell death) after 48 h’ treatment as mean ± SEM of at least three independent experiments each performed in quadruplicate

Regardless UAE derived extracts, we only tested Yarrow UAE (Ethanol) from the OVM, as it has been shown to be the most effective UAE extracts tested in MIA PaCa-2. As shows Fig. 3b, IC50 value was much higher in PANC-1 compared to MIA PaCa-2 value (Fig. 2b).

In the other hand, we compared the effect on cell viability of commercial extracts obtained industrially by glycerin tincture, with Yarrow and Marigold SFE extracts and Yarrow UAE (ethanol) OVM extract. As shown in Tables 1 and 2, commercial extracts from Yarrow or Marigold did not exerted any effect on cell viability of MIA PaCa-2 and PANC-1 in the range of the doses tested (Fig. 2f and Fig. 3c, respectively) (GI50, TGI and LC50 values were higher than 500 μg/mL).

Finally, we have determined the effect on cell viability in a non-tumoral cell line (Additional file 1: Table S1, Additional file 1: Figure S1) to ensure the nontoxic response. The IC50 in the non-tumoral cells is 81,6 ± 14,1 μg/mL for Yarrow and 84,3 ± 21,2 for Marigold SFE extract.

### Yarrow and Marigold SFE extracts induce pancreatic cancer cell death through apoptosis

To assess the mechanism by which Yarrow and Marigold SFE extracts induce cell growth inhibition, we have analyzed caspase 3/7 activation and the apoptotic populations.

**Fig. 4:**
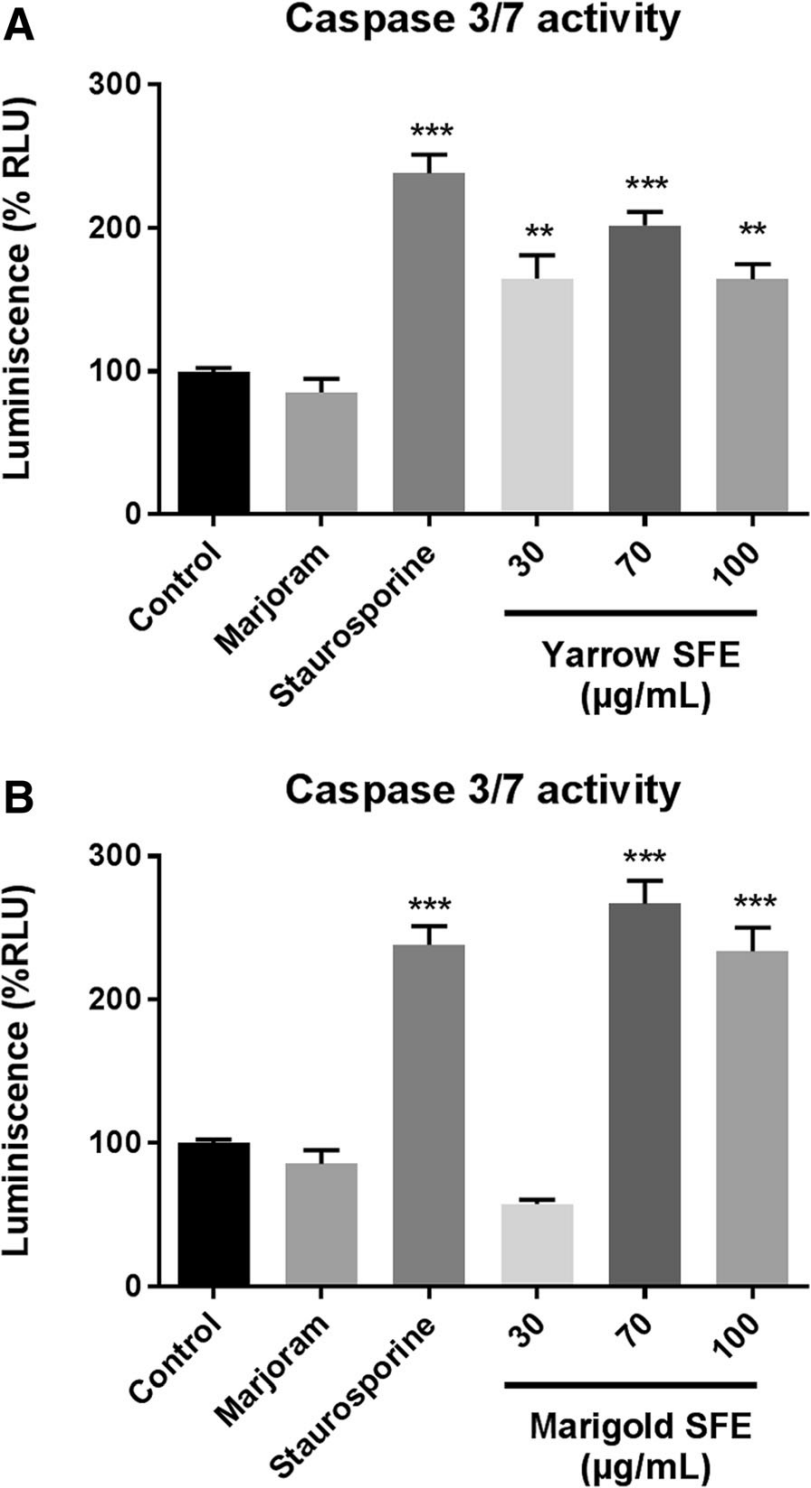
Apoptosis triggered by treatment with Asteraceae plant extracts on MIA Paca-2 cells. Activation of caspase 3 and 7 after 48 h treatment with Yarrow SFE extracts (**a**) or with Marigold SFE extracts (**b**), with the positive (Staurosporine 1,5 μM) and negative (Marjoram SFE 100 μg/mL) controls. Data represent means ± S.E.M of three independent experiments each performed in duplicate. Asterisks indicate statistical differences in treated cells with respect to the control (non-treated cells, DMSO 0.1%). **p < 0.01; ***p < 0.001

Firstly, and as shown in Fig. 4, after 48 h of treatment, both Yarrow and Marigold SFE increased the levels of active (cleaved) caspases 3/7.

**Fig. 5:**
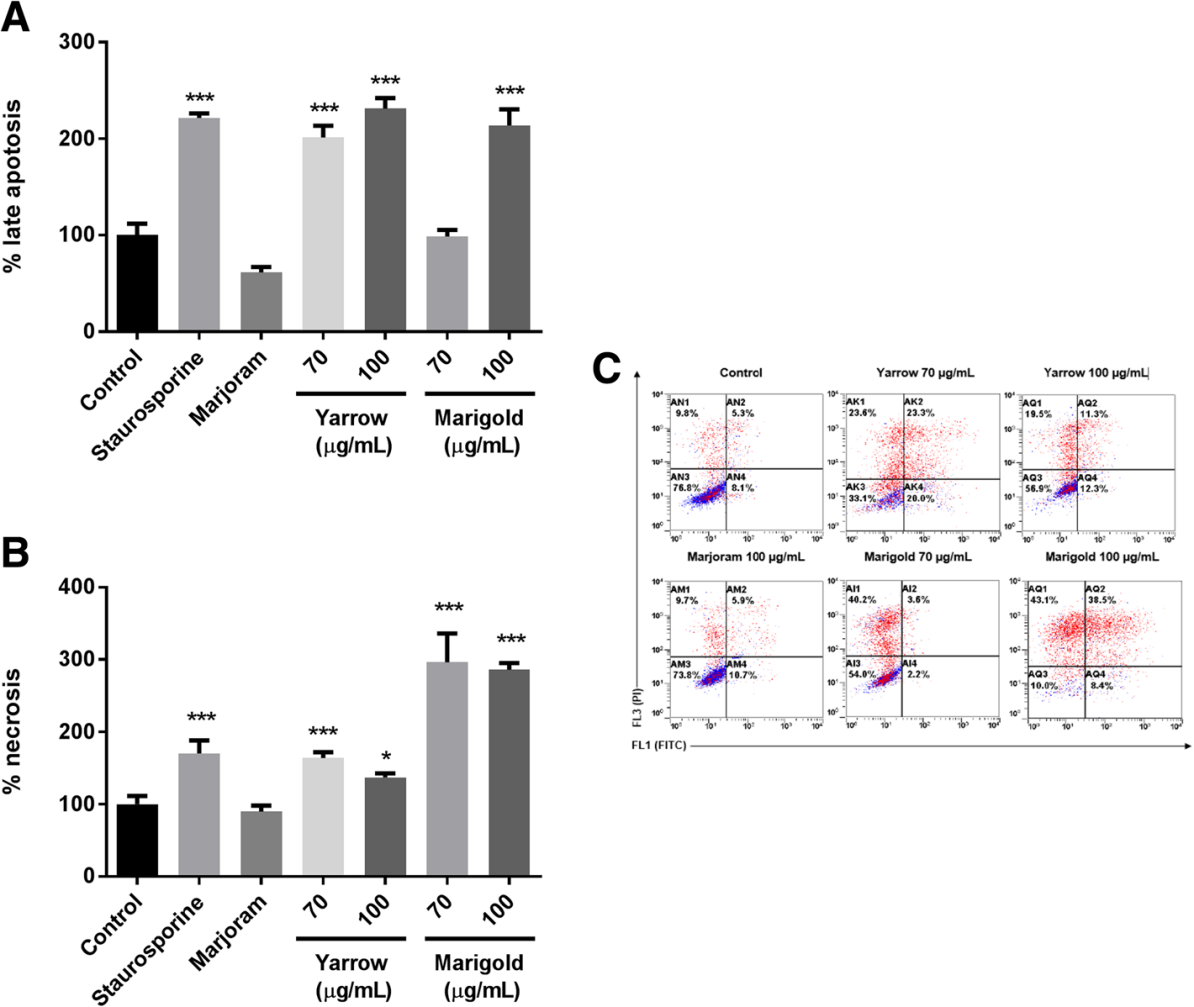
Different phases of cell death after 24 h treatment with Asteraceae plants on MIA Paca-2. The % of apoptotic cells depending on Yarrow and Marigold SFE extracts (**a**) and the % of necrotic populations after those treatments (**b**). A representative flow cytometry diagram (**c**). Annexin V-FITC/PI double staining discriminates the live cells (Annexin V−/PI−; bottom left quadrant), early apoptotic cells (Annexin V+/PI−; bottom right quadrant), late apoptotic (Annexin V+/PI+; upper right quadrant), and necrotic or dead cells (Annexin V−/PI+; upper left quadrant). Data represent means ± S.E.M of at least four independent experiments each performed in triplicate. Asterisks indicate statistical differences in treated cells with respect to the control (non-treated cells, DMSO 0.1%). *p < 0.05; ***p < 0.001

In addition, we have also determined, by flow cytometry with Annexin-V and PI staining, the distribution of apoptotic and necrotic cell subpopulations. Fig. 5a shows percentages of early apoptotic cells (PI−/Annexin V+), late apoptotic cells (PI+/Annexin V+) and necrotic cells (PI+/Annexin V-). Both extracts induced late apoptosis at 100 μg/mL (Fig. 5a). Interestingly Marigold gave rise to a higher percentage of necrotic cells (Fig. 5b). Fig. 5c shows a representative flow cytometry plot indicating the gates for the different subpopulations.

### Yarrow and Marigold SFE extracts inhibit colony growth in 3D

We were interested in determining if Yarrow and Marigold SFE extracts affect the ability of MIA PaCa-2 epithelial cancer cells to form colonies when grown in 3D culture.

**Fig. 6:**
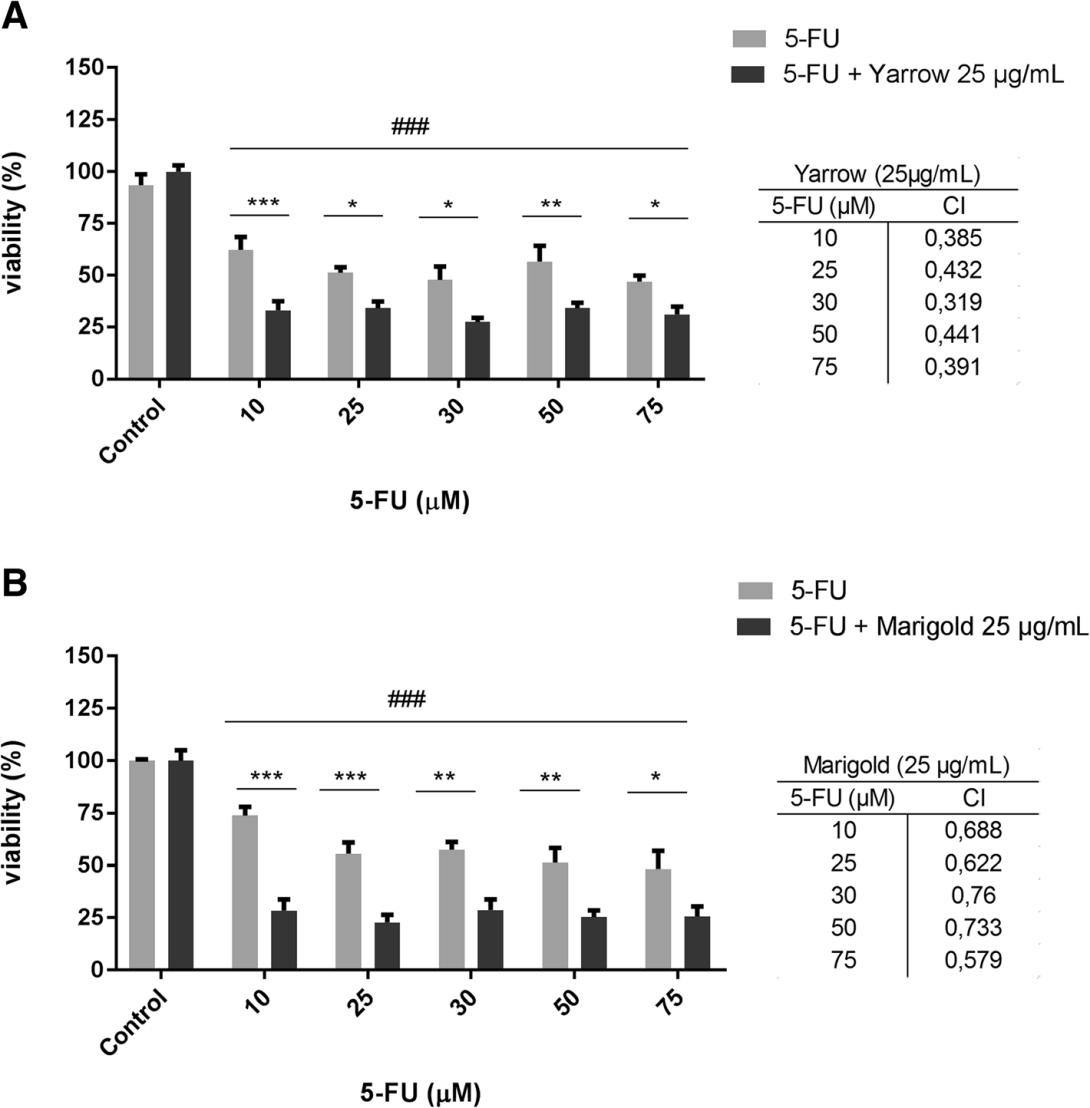
MIA PaCa-2 response and Combination Index (CI) when cells were pre-treated 8 h with A) Yarrow or B) Marigold, followed by an exposure of increased doses of 5-FU for 72 h. Data represent means ± S.E.M of at three independent experiments each performed in quadruplicate. Asterisks (*) indicate statistical differences between 5-FU and Yarrow/Marigold+ 5-FU treatments *p < 0.05; **p < 0.01 ***p < 0.001. # indicates statistical differences in treated cells with respect to the control (DMSO 0.1%). ### *p* < 0.001

We first plated MIA PaCa-2 in Matrigel and 72 h later, when the colonies were formed, we treated the cells with 30, 50 and 70 μg/mL of the extracts, and the control with DMSO (0.1% *v*/v) (Fig. 6a). Yarrow reduced the size and number of the spheres maintaining their integrity (Fig. 6b), while Marigold affected significantly both parameters when cells were treated with 70 μg/mL (Fig. 6c), promoting the disruption of colonies (Fig. 6a). Both extracts diminished colony integrity suggesting that stem or progenitor cells are not able to sustain the tumor in vivo when treated with Yarrow or Marigold.

### Yarrow and Marigold SFE extracts exhibit synergizes with 5-FU treatment

**Fig. 7:**
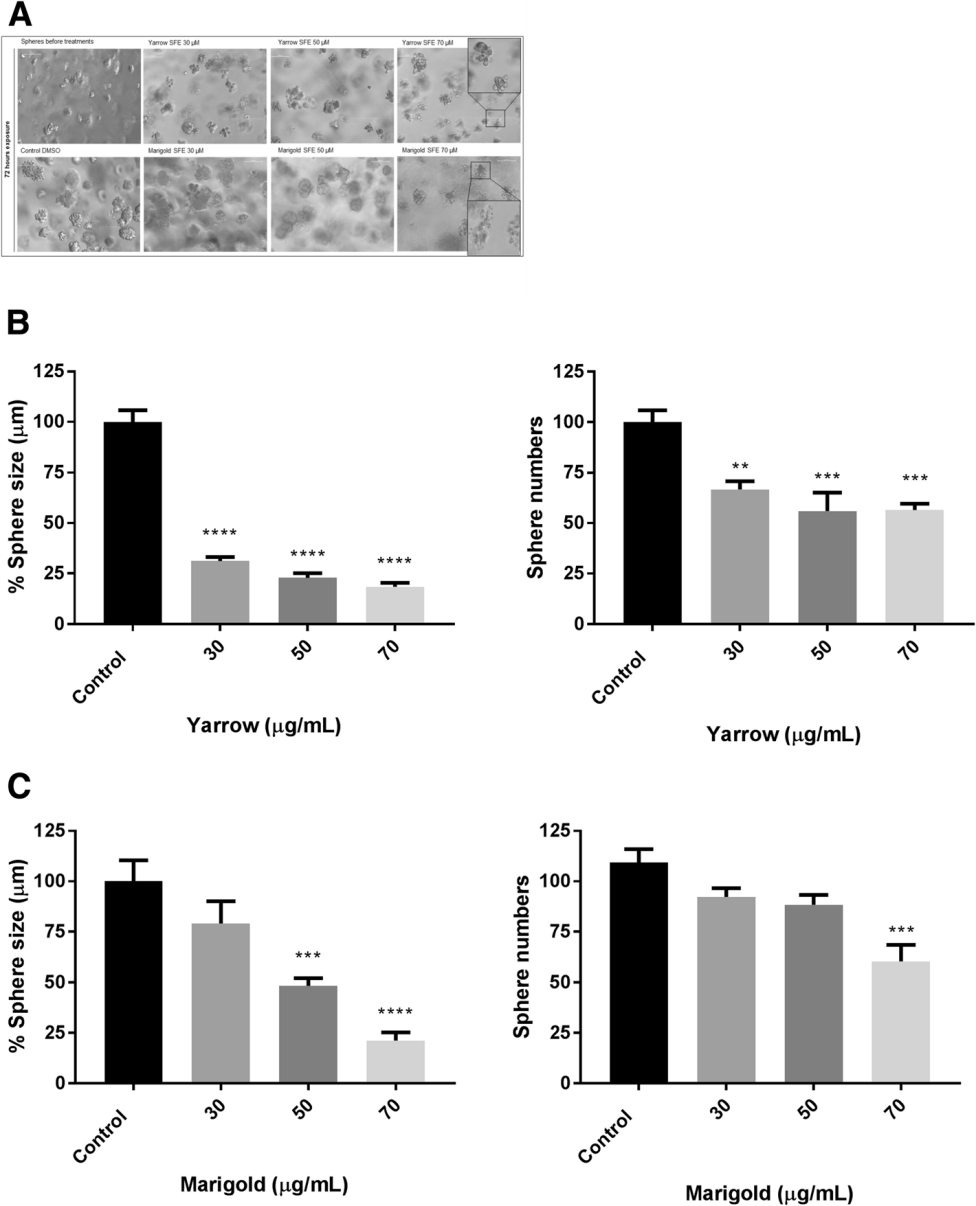
Tridimensional MIA PaCa-2 cells when exposed to Asteraceae Extracts. **a**) Representative images of spheres after Yarrow (up) and Marigold (down) treatments for 72 h. Sphere number, size and morphology after Yarrow (**b**) and Marigold (**c**) exposures. Data represent means ± S.E.M of three independent experiments each performed in duplicate. Asterisks indicate statistical differences in treated cells with respect to the control (DMSO, 0.1%) ***p < 0.001; ****p < 0.0001

Finally, we analyzed the effect of Yarrow SFE and Marigold SFE in combination with the antitumoral drug 5-fluororacil (5-FU) on MIA PaCa-2, which is usually proposed in clinic to treat pancreatic cancer. Cells were treated with different concentrations of 5-FU for 72 h. We have observed that both Yarrow and Marigold markedly potentiate the antiproliferative effect of 5-FU when cells were pre-treated with these extracts, showing a higher cytotoxic effect when combining a plant extract with the antimetabolite 5-FU (Fig. 7), with a significant decrease in cell viability. Thus, according to the Chou–Talalay method [16], the combination of Yarrow with 5-FU and the combination of Marigold with 5-FU resulted in a synergistic effect displaying a (combinatory index) CI value < 1 in every combination assayed (Fig. 7).

## Discussion

In this work, we have evaluated the antitumoral properties of four plants (Yarrow, Marigold, Balm and Marjoram) derived extracts in pancreatic cancer cell lines.

Importantly, two different green technologies have been used to obtain the bioactive components from these plants: Supercritical Fluid Extraction (SFE) and Ultrasonic Assisted Extraction (UAE). For UAE extractions, two different raw materials were compared -the Residual Vegetable Material obtained after the SFE step (RVM), and the Original Vegetable Material (OVM)-.

Yarrow and Marigold extracts obtained by SFE were the most effective ones on inhibiting cell proliferation, exhibiting similar values of IC50, TGI and LC50 in MIA PaCa-2 cells (Table 1). These results are within the range of those previously reported for Marigold extract obtained by Laser extraction in pancreatic cancer, leukemia and fibrosarcoma (IC50 = 60 μg/mL) [12]; or by those obtained by ethyl alcohol maceration in melanoma (IC50 = 50 μg/mL) [17].

Yarrow extracts obtained by UAE (Ethanol) from OVM and RVM were also effective on inhibiting cell viability. The concentration needed to achieve 50% of growth inhibition (GI50 = 49,4 ± 25,7 μg/mL) is within the interval of that obtained in other works by methanolic stirring extraction in hepatocellular (39.02 ± 2.9 μg/mL) and cervical carcinomas (47.1 ± 1.8 μg/mL) models [18].

Conversely and related to Lamiaceae family plants (Balm and Marjoram), none of the extracts (nor SFE, nor UAE) had any effect on the range of concentration tested (Table 1, Fig. 2). Although it has been described ethanolic Marjoram extract to promote lymphoblastic leukemia cell death [19], and Balm extract in hepatocellular and gastric carcinomas [20], in both cases the range of concentrations were around one thousand times higher than the ones tested in this work (IC50 = 8 mg/mL for Marjoram and around 70 mg/mL for Balm).

Thus, Marigold and Yarrow SFE extracts displayed better dose-dependence activity compared to the UAE and other extraction methodologies, both in MIA PaCa-2 and in the more resistant model, PANC-1. Indeed, here, Yarrow and Marigold SFE extracts have a IC50 dose much higher in a colon non-cancer cell line (CCD18), comparing the IC50 in a colon cancer cell line (SW-620) (Additional file 1: Figure S1, Additional file 1: Table S1), which supports a therapeutic window for the use of the extracts in combination with current therapy.

In the other hand, current treatment in clinic is mostly based on 5-fluorouracil (5-FU) and gemcitabine, even combined with other drugs. In this sense, we wanted to determine a possible synergism of Yarrow and Marigold SFE extracts with 5-FU. Thus, when pre-conditioning MIA PaCa-2 cells with Yarrow or Marigold, the cytotoxic effect mediated by the subsequent 5-FU exposure has been increasing (Fig. 7). This enhancement in 5-FU effect has been previously described for other SFE extracts, such as Rosemary in colon cancer model [21], but Yarrow and Marigold are here described for the first time. In this sense, this synergism could be considered a promise as a co-adjuvant treatment, improving patient survival, given that both extracts potentiate the effect of a first-line chemotherapeutic agent. Until date, plants extracts, as a grouped set of molecules, have been tested and used to reduce chemotherapy side effects and for improve patient’s quality of life [22], with few approaches proposing their use as a second-line therapy [23]. Thus, there is a need to promote clinical trials with plant extracts, as an ensemble of phytochemicals, after validating the antiproliferative hypothesis in murine models.

Further describing the effect of Yarrow and Marigold SFE extracts, and regarding their mechanism of action, we have found that both extracts induce apoptosis through caspase 3/7 cleavage (Fig. 4), although Marigold has also shown to promote the accumulation of high levels of a necrotic cell population. These results suggest that different signaling pathways may be implicated in the induction of cell death for each extract. In this context, restoring apoptotic signaling pathways has been proposed as a strategy for cancer treatment. In fact, most of the antitumoral agents exert their effect through induction of apoptosis [24, 25]. Particularly, these results are in accordance with those described previously for Marigold [12] related with programmed cell death. Concerning Yarrow, there are few studies which describe its involvement in the induction of apoptosis, contributing this study to the first evidences of them.

Finally, we also have demonstrated that Yarrow and Marigold SFE inhibit transforming activity (Fig. 7). Growing and giving rise to 3D colonies is a way to monitor cell malignancy and the ability of stem and progenitor cells to sustain the tumor. If the proposed extracts affect the colony formation, cells are not able to evade signals that restraint their growth. In this sense, herein, we have seen that Yarrow induces a dose-dependent decrease in cell transformation and brakes the 3D sphere growth, while, Marigold induces disruption of the integrity of 3D colonies that may be due to the loose of their mesenchymal phenotype associated with an increased motility.

These results also suggest differences in the specific antitumoral mechanism of both extracts. They demonstrate that even both plants are from the same family (Asteraceae), there are differences in the way they exert their bioactive effect against pancreatic tumor cells. These differences encourage further studies to better understand the molecular action of the extracts, conducting individual approaches for each plant type. Once we a deeper understanding of their anticancer profile reached, the extracts could be registered for this purpose, beyond their current use as herbal preparation in most of the European Union countries.

## Conclusion

This work has demonstrated that Yarrow and Marigold supercritical fluid extracts (SFE), besides avoiding chemical agents for their obtaining, allow to get effective amounts of antitumor phytochemical agents with effects not only in cell viability and 3D grown, but also in sensitizing tumor cells to chemotherapeutic agents such as 5-FU.

Related to Marjoram and Balm, their derived extracts have shown no cytotoxic effect in the pancreatic cell lines tested.

## Additional file

Additional file 1: **Table S1.** Sensitivity of SW-620 human colon cancer cells and CCD18 colon cells to Yarrow and Marigold extracts. Concentrations required to achieve the IC50, LC50, IG50 and TGI of Yarrow and Marigold SFE extracts treatments in colorrectal derived cancer cells and non tumoral colon cells. **Figure S1.** Dose-response curves of cell viability assays after 48 h treatment of SW-620 colon cancer cells (A) versus CCD18 non-cancer cells (B) with increasing concentrations of Yarrow and Marigold SFE extracts. Dose-response curves of cancer and non-cancer colon cells after treatments with Yarrow and Marigold SFE extracts. (DOCX 151 kb)

## Abbreviations

DMSO: Dimethyl Sulphoxide
GI50: concentration to induce 50% growth inhibition
IC50: concentration to induce 50% cell viability inhibition
LC50: lethal concentration
OVM: Original Vegetable Matrix
RVM: Residual Vegetable Matrix
SFE: Supercritical Fluid Extraction
TGI: concentration to induce total growth inhibition
UAE: Ultrasound Assisted Extraction
