# Supplementary material for: Identification of antitumoral agents against human pancreatic cancer cells from Asteraceae and Lamiaceae plant extracts
Source: BMC Complement Altern Med. 2018 Sep 17;18:254. doi: 10.1186/s12906-018-2322-6 (PMC6142333; doi:10.1186/s12906-018-2322-6)
Supplement: Supplementary file 1 — Table S1. Sensitivity of SW-620 human colon cancer cells and CCD18 colon cells to Yarrow and Marigold extracts. Concentrations required to achieve the IC50, LC50, IG50 and TGI of Yarrow and Marigold SFE extracts treatments in colorrectal derived cancer cells and non tumoral colon cells. Figure S1. Dose-response curves of cell viability assays after 48 h treatment of SW-620 colon cancer cells (A) versus CCD18 non-cancer cells (B) with increasing concentrations of Yarrow and Marigold SFE extracts. Dose-response curves of cancer and non-cancer colon cells after treatments with Yarrow and Marigold SFE extracts. (DOCX 151 kb) [file 12906_2018_2322_MOESM1_ESM.docx]

**Additional material**

Methodology: non-cancerous colon cells (CCD18) were cultured in EMEM supplemented with 10% FBS. Cells were kept under standard conditions of temperature (37°C), humidity (95%) and carbon dioxide (5%). The cytotoxicity assays were carried out through MTT assay, as it has been done for the cancerous cells (MIA PaCa-2, PANC-1).

For cancerous colon cells (SW-620), the conditions and experimentation were the same than for pancreatic cancer cells, as explained in Methods section.

**Table S1**. Sensitivity of SW-620 human colon cancer cells and CCD18 colon cells to Yarrow and Marigold extracts

| **Extract** | **Parameter (µg/mL)** | **SW-620** | **CCD18** |
| --- | --- | --- | --- |
| **Yarrow** | IC50 | 49,8 ± 1,6 | 81,6 ± 14,1 |
|  | GI50 | 30,1 ± 2,3 | >150 |
|  | TGI | 90,6 ± 3,7 | >150 |
|  | LC50 | >150 | >150 |
| **Marigold** | IC50 | 63,4 ± 3,0 | 84,3 ± 21,2 |
|  | GI50 | 59,06 ± 2,6 | >150 |
|  | TGI | >100 | >150 |
|  | LC50 | >100 | >150 |

^Data are the mean ± SEM of at least three independent experiments each performed in triplicate.^

^(IC50) Effective concentration required for 50% inhibition of cell proliferation, after 48h treatment.^

^(GI50) Concentration required for 50% cell growth inhibition, after 48h treatment.^

^(TGI) Concentration required for total cell growth inhibition, after 48h treatment.^

^(LC50) Concentration required for 50% cell death, after 48h treatment.^


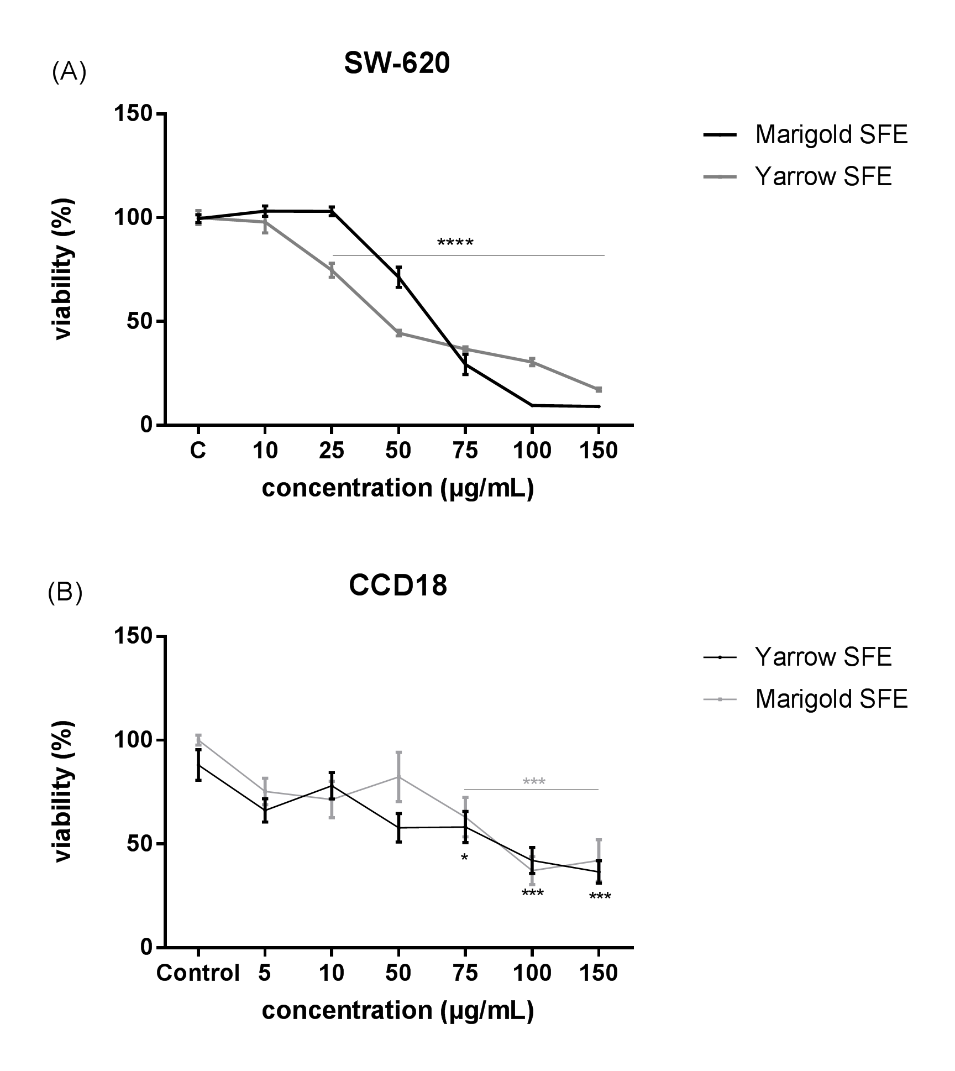


**Fig. S1**. Dose-response curves of cell viability assays after 48 hours’ treatment of SW-620 colon cancer cells (A) versus CCD18 non-cancer cells (B) with increasing concentrations of Yarrow and Marigold SFE extracts. Data represent means ± S.E.M of at least three independent experiments each performed in quadruplicate. Asterisks indicate statistical differences in treated cells with respect to the control (non-treated cells, DMSO 0.1%). ***p<0.001; ****p<0.0001.
